# The relative importance of work-related and non-work-related stressors and perceived social support on global perceived stress in a cross-sectional population-based sample

**DOI:** 10.1186/s12889-021-10594-2

**Published:** 2021-03-19

**Authors:** Jes Bak Sørensen, Mathias Lasgaard, Morten Vejs Willert, Finn Breinholt Larsen

**Affiliations:** 1 DEFACTUM, Central Denmark Region, Olof Palmes Alle 15, DK-8200 Aarhus N, Denmark; 2 Department of Occupational Medicine, Danish Ramazzini Centre, Aarhus University Hospital, Aarhus, Denmark

**Keywords:** Stress, Perceived stress scale, Survey, Population-based, Stressors, Work, Non-work, Social support, Dominance analysis

## Abstract

**Background:**

High levels of perceived stress have a negative bearing on health and well-being, and stress is a major public health issue. According to the Stress Process Model, stressors are socially patterned and combine to produce strain. Despite this, most studies on stress have focused on work-related stressors leaving non-work determinants under-investigated. The aim of the present study was to determine the relative importance of work-related and non-work-related stressors and perceived social support for the overall perceived stress level.

**Methods:**

Self-reported data were drawn from the 2017 population-based health survey “How are you?” conducted in the Central Denmark Region (*N* = 32,417). Data were linked with data drawn from national administrative registers. Work- and non-work-related stressors assessed included major life events, chronic stressors and daily hassles. Perceived social support was assessed using a single question. Overall perceived stress was assessed by the 10-item Perceived Stress Scale. We conducted dominance analyses based on a multiple linear regression model to determine the most important explanatory variables of overall perceived stress. Analyses were weighted and adjusted.

**Results:**

Work- and non-work-related stressors along with perceived social support explained 42.5% of the total variance (R^2^) in overall perceived stress. The most important explanatory variables were disease, perceived social support and work situation. The stratified analyses produced slightly varying results (“dominance profiles”) of perceived stress between subgroups. Work situation was the most important explanatory variable in the employed group. However, adding non-work-related explanatory variables to the analysis tripled the explained variance.

**Conclusions:**

The overall level of perceived stress can be statistically explained by a combination of work- and non-work-related stressors and perceived social support both at population level and in subgroups. The most important explanatory variables of overall perceived stress are disease, perceived social support and work situation. Results indicate that public health strategies aiming to reduce stress should take a comprehensive approach and address a variety of stressor domains rather than focus on a single domain.

**Trial registration:**

The study was approved by the Danish Data Protection Agency (r. no. 2012-58-0006) and registered in the Central Denmark Region (r. no. 1-16-02-593-16).

**Supplementary Information:**

The online version contains supplementary material available at 10.1186/s12889-021-10594-2.

## Background

Stress is a major public health issue and has long been a topic attracting much interest across a wide range of research fields [1–3]. A particularly active area of research has been work-related stress [2, 4, 5]. This area has predominantly been inspired by the Demand-Control-Support [6] and Effort-Reward-Imbalance [7] models, focusing on the interplay between the psychosocial work environment and workers’ mental health. With the exception of work-life balance, most research in this area has analysed the workplace as a closed system where high levels of stress among employees are linked primarily to occupational stressors thereby leaving non-work determinants under-investigated [3, 8].

In the present study, we examine the relative importance of work-related and non-work-related stressors and perceived social support for statistically explaining the overall level of perceived stress. As a theoretical framework for the study, we combine two pivotal stress research models: the psychological Transactional Stress Model [9] and the sociological Stress Process Model [10]. In doing so, we focus on stress as a mental or emotional reaction to socially patterned exposures.

We use the definition of stress by Lazarus and Folkman in which stress is a state of emotional strain and pressure arising in an individual when demands in the environment are perceived as “taxing or exceeding his or her resources and endangering his or her wellbeing” [9]. Key concepts are cognitive appraisal and coping. While appraisal mechanisms (i.e., primary, secondary and re-appraisal) affect the extent to which the situation is perceived as stressful, coping process (i.e., how the individual handle the stressful situation) determine whether the stress will be alleviated or continue. High levels of perceived stress have a negative impact on health and well-being [1]. Furthermore, perceived stress increases the risk of unemployment [11], and long-term exposure to stressors is a risk factor for developing mental and physical diseases including depression [12–15] and anxiety disorders [16], substance abuse [17, 18], coronary heart disease [19, 20], type 2 diabetes [21, 22] and metabolic syndrome [23]. Consequently, high levels of perceived stress are associated with an increased risk of premature death [24].

While the Transactional Stress Model focuses on cognitive processes that occur in the interaction between the individual and the environment, the Stress Process Model introduced by Pearlin and colleagues [10] focuses on social phenomena that cause stress (termed stressors). Pearlin recognized that stressors are socially patterned and rarely act in isolation but combine in an additive or multiplicative manner to produce strain [10]. Thus, the Stress Process Model complements the Transaction Stress Model and points to social causes of stress that could potentially be changed.

The Stress Process Model has served as a paradigm for research into an expanding range of stressors [25]. Pearlin argued strongly that stress research should address the full array of stressors found in a person’s life rather than single stressors in isolation [25, 26]. There is, however, no clear definition of “the full array of stressors”, but Pearlin points towards both chronic strains and life events [26], and Wheaton adds daily hassles to the list [27]. Despite this, the understanding of the complexity of the stress process remains deficient in the empirical literature [28]. According to a review of trends in stress research, literature on specific stressors has become more abundant and research has become increasingly segmented [29]. This trend may result in a return to pre-Stress Process Model approaches addressing one stressor at a time and thus ignoring the additive or multiplicative effect of stressors.

One of the gains of the extensive sociological, psychological and epidemiological research into stressors is a conceptual division of the stress continuum into life events, chronic stressors and daily hassles [26, 27]. “Life events” are sudden, discrete occurrences such as the death of a near relative or being laid off from work unexpectedly. “Chronic stressors” are enduring strains such as discrimination, permanently compromised health or conflicting demands of multiple social roles. “Daily hassles” include episodic – expected or unexpected – minor events which, as they accumulate, may constitute stressors such as a parent and a child struggling over bedtime or delays in morning traffic. In addition, these very diverse stressors vary in respect to duration, severity and the context in which they occur. It is acknowledged that these different types of stressors all contribute to the overall burden of stress in the population [27]. Thus, the measures used for exposure to stressors should tap into a broad spectrum from discrete life events to chronic stressors and daily hassles. The consequences of chronic or recurring stressors may be particularly severe when they surface within major social domains, such as breadwinning, job and family [30]. A better understanding of domain-specific stressors and their relative contribution to the overall perceived burden of stress is important for effective public health strategies [31, 32]. Prevention and reduction of stress at population level and in target groups facing special needs should be based on empirical knowledge of the main sources of stress.

The other major component of the Stress Process Model is resources available to cope with the challenges experienced [33]. Central to this component is access to social support from one’s surroundings. In particular, empirical research has established that it is not the actual availability of support that promotes coping but its perceived availability [28, 34]. It has been theorised that social support buffers the effects of stressors [10, 26, 35]. However, a review indicates that the stress-buffering effects of social support are “less dramatic and consistent” than the direct effects of social support on mental health [36]. This underscores the relevance of investigating the direct effect of perceived social support on the overall stress burden on par with stressors within various social domains.

In this study, we return to a comprehensive approach to stress suggested by Pearlin [30] by investigating the relative importance of perceived stressors occurring within major social domains and perceived social support as explanatory variables of perceived stress in a general adult population.

### Aim

The aim of the study was to determine the relative importance of work-related and non-work-related stressors and perceived social support as explanatory variables of the overall perceived stress level. The study examines perceived stressors and perceived social support at population level and in socio-demographic subgroups in order to generate knowledge for public health strategies.

## Methods

### Study design and data collection

Self-reported data were drawn from the 2017 population-based health survey “How are you?” conducted in the Central Denmark Region. In 2017, approximately 23% of the Danish population were living in the Central Denmark Region, and the demographic composition (gender, age and educational attainment) of the population was similar to that of the total Danish population [37]. The survey included a representative population sample of 52,000 citizens aged 16 years and above drawn from the Danish Civil Registration System [38]. Participants were invited to complete a web-based or postal questionnaire [39]. Those who failed to respond to the survey received up to four reminders. The response rate was 62% (32,417), and 80% of the respondents completed the web questionnaire. The study was approved by the Danish Data Protection Agency (r. no. 2012-58-0006) and the Central Denmark Region (r. no. 1–16–02-593-16). Each participant received written information about the purpose of the survey, and informed consent was obtained from all subjects. All methods were carried out in accordance with relevant guidelines and regulations along with the approval. Data from the survey were linked with national administrative registers using the unique personal identification number assigned to all Danish citizens [38]. Register data included age, gender, ethnic background and labour market status.

### Variables

Outcome (Perceived Stress Scale) and exposure (perceived stressors and perceived social support) were selected according to the Transactional Stress Model in which a person’s assessment of a potential stressor as benign, neutral or threatening serves as the link between loads and demands in the environment and the emotional strain experienced by the person [9]. The selected stressors include a wide range of life domains in order to examine how much each contributes to the global stress level. Furthermore, in the Stress Process Model, stressors and social support constitute the major components causing stress [10].

#### Perceived stress scale

The level of perceived stress was assessed by the 10-item Perceived Stress Scale (PSS) [40]. Based on Lazarus’ stress model [41, 42], the PSS is a global measure of stress in which stress relies upon the person’s perception of the stressor as stressful or not. The ten items ask how often in the past month life was appraised as unpredictable, uncontrollable and overloaded (e.g., “In the last month, how often have you been upset because of something that happened unexpectedly?”) [40]. The items were scored from 0 to 4 (“never”, “almost never”, “sometimes”, “fairly often” or “very often”). An increasing sum score (range 0 to 40) indicates an increasing perceived stress level [40]. PSS does not have cut-off values for determining high or low levels of perceived stress [40]. However, a number of studies have proposed cut-off values ranging from 15 to 18 for high levels of perceived stress [11, 24, 43, 44]. PSS has satisfying psychometric properties [45, 46] and has been used in a number of population-based studies (e.g. [11, 43, 47–49]). In the present study, Cronbach’s α indicated that the scale had a high internal consistency (α = 0.87).

#### Perceived stressors

Work-related and non-work-related perceived stressors were assessed using nine questions covering seven categories from the Life Event Questionnaire [50]. The selection of questions was inspired by The Danish National Birth Cohort [51–53]. The questions cover major life events, chronic stressors and daily hassles. Perceived stressors were assessed asking “Within the past 12 months, have you felt burdened by some of the following things?” The questionnaire covers work situation, financial circumstances, housing conditions, relationship with partner, relationship with family and friends, own disease, disease among close relatives, deaths among close relatives and other types of burdens. The response categories were “no”, “yes, a little”, “yes, partly” or “yes, a lot”. In the present study, all four response categories of each variable were used in the analyses.

The survey also includes a more elaborate questionnaire on work that covers quantitative demands, job influence, satisfaction and recognition, work-life conflicts, physical and emotional wear and tear, and physical load [54]. However, a previous study found that including specific aspects of psychosocial work conditions did not provide additional information than a single global question [55]. In line with this, preliminary analyses using the more elaborate questionnaire did not alter our results. Thus, in order to keep the model simple, the single question about work situation was preferred.

#### Perceived social support

Perceived social support was assessed using a single item: “Do you have anyone to talk to if you have problems or need support?” The response format was “yes, always”, “yes, mostly”, “yes, sometimes” or “no, never or almost never”. In the present study, all four response categories of each variable were used in the analyses. The question was inspired by The MOS Social Support Instrument [56].

#### Socio-demographic variables

Age and gender were assessed using a combination of self-reported and register data. Ethnic background was defined using the Danish Civil Registration System [38]. Educational attainment was self-reported and categorised as low (primary school, no further education), medium (upper secondary education, vocational education and/or short higher education) or high (bachelor’s degree or higher level of education) according to the Danish version of the International Standard Classification of Education [57]. Students were categorised according to their expected graduation level. Labour market status was assed using a combination of self-reported data and data from the Danish Register for Evaluation of Marginalization [58].

### Data analysis

We conducted dominance analysis, a relatively new computer-intensive method, to determine the most important explanatory variables of perceived stress in the population and in stratified analyses (i.e., gender, age, educational attainment and labour market status) [59, 60]. The analyses were based on a multiple linear regression model with PSS as the dependent variable and the nine different stressors and perceived social support as explanatory variables. Dominance analysis is a method of investigating importance and ranking of explanatory variables (“dominance profiles”) according to how much each variable contributes to the total variance of the dependent variable in a model [59]. The method is particularly suitable when explanatory variables are intercorrelated, as may be expected in this study. Dominance analysis is an ensemble method based on estimation of all possible regression models (all subset regressions) [61]. The average increase in total variance explained by the model (*R*^2^) when adding a variable to all possible sub-models quantifies the importance of the explanatory variable. Dominance analysis is used for decomposition of the total R^2^, but it has rarely been used in stress research [62]. The dominance analyses in the present study consisted of 1023 (2^10–1) regression models containing all possible combinations of explanatory variables. In addition to the ten explanatory variables, gender, age, ethnic background and educational attainment were included in all models.

Perceived stress and the perceived stressors and social support are collected at the same time, and are likely to affect each other. This is foreseen since stressors are socially patterned and combine to produce strain [10]. This does not pose a problem, since we use dominance analysis to determine the relative importance of perceived stressors and perceived social support as explanatory variables of PSS [60].

To reduce sampling and non-response bias, weights constructed by Statistics Denmark using a model-based calibration approach and including socio-demographic characteristics, income, social benefits and healthcare utilisation were applied [39, 63].

Prior to the analyses, data were screened for missing values. The percentage of missing values was acceptable (0–13%). Missing PSS items ranged from 4.3% (item 1) to 5.1% (item 8). If one, two or three items of the PSS scale were missing, the mean of the available items was used to calculate the scale score [64]. If responses to more than three items were missing, the PSS score was regarded as missing. Hence, 1392 observations (4.3%) were excluded.

For stressors, missing values were treated as “no” if respondents had answered at least one of the nine stressor questions. Hence, 26,799 respondents (83%) had complete stressor data, 3462 respondents (11%) had partially complete stressor data and 2156 respondents (7%) were missing. A similar approach was adopted in the “How are you?” health survey with regards to measures of chronic diseases [65].

PSS was obtained in 95.7% of the respondent (Table 1), and stressors were obtained in 93.3% of the respondents (Table 2). Respondents with missing PSS or stressors were more likely to be 16–24 years old, have an ethnic background other than Danish and an unknown educational attainment or work situation (results not shown).

**Table 1:** Characteristics of respondents with perceived stress scale (PSS) score (*N* = 31,025)

	PSS mean (SD)	N	%^**a**^
**All**	12.2 (7.2)	31,025	–
**Gender**			
Men	11.4 (6.7)	14,447	49.7
Women	13.1 (7.6)	16,578	50.3
**Age**			
16–24	14.2 (6.5)	3505	14.8
25–64	12.2 (7.2)	19,122	62.7
≥ 65	11.2 (7.5)	8398	22.5
**Ethnic background**			
Danish	12.0 (7.4)	29,271	89.9
Other Western^b^	13.4 (5.3)	784	4.2
Non-Western^c^	15.6 (5.2)	970	5.9
**Educational attainment**			
Low (0–10 years)	14.4 (7.4)	4437	15.2
Medium (11–15 years)	12.0 (7.3)	16,923	51.8
High (15- years)	11.2 (6.6)	8497	28.3
Unknown	14.5 (6.5)	1168	4.7
**Employment status**			
Students	13.7 (6.2)	2546	11.2
Employed	11.1 (6.7)	16,288	51.1
Unemployed	15.9 (7.2)	539	2.0
Cash or sickness benefits, etc.	17.9 (7.8)	1414	5.6
Disability pension	17.8 (7.0)	1086	4.1
Early retirement pension	9.7 (7.0)	730	2.0
Retirement pension	11.4 (7.4)	7576	20.7
Unknown	13.7 (6.6)	846	3.3

^a^Weighted percentage

^b^Other Western countries: all 27 EU countries, Andorra, Iceland, Liechtenstein, Monaco, Norway, San Marino, Switzerland, Vatican State, Canada, USA, UK, Australia and New Zealand

^c^Non-Western: all other countries

**Table 2:** Prevalence of stressors and perceived social support (*N* = 30,261)

		PSS mean (SD)	N	%^**a**^
**Financial circumstances**				
No		10.5 (6.9)	19,120	58.9
Yes, a little		12.7 (6.4)	7121	25.2
Yes, partly		15.8 (6.5)	2305	9.0
Yes, a lot		19.5 (6.9)	1715	6.9
**Housing conditions**				
No		11.1 (7.0)	24,527	77.2
Yes, a little		14.1 (6.6)	3616	13.8
Yes, partly		17.1 (6.4)	1279	5.3
Yes, a lot		19.6 (6.7)	838	3.6
**Work situation**				
No		10.7 (7.0)	18,923	59.8
Yes, a little		12.1 (6.2)	6587	22.9
Yes, partly		15.6 (6.3)	2708	9.7
Yes, a lot		19.3 (6.8)	2040	7.7
**Relationship with partner**				
No		11.3 (7.0)	22,927	75.1
Yes, a little		13.6 (6.9)	5196	17.1
Yes, partly		16.6 (6.9)	1366	4.9
Yes, a lot		19.2 (6.9)	768	3.0
**Relationship with family and friends**				
No		10.7 (6.7)	22,639	72.9
Yes, a little		14.8 (6.9)	6030	21.1
Yes, partly		19.3 (6.8)	1174	4.4
Yes, a lot		22.3 (7.0)	413	1.7
**Disease**				
No		10.1 (6.3)	17,252	57.8
Yes, a little		12.9 (6.7)	7781	24.7
Yes, partly		16.4 (6.9)	3151	10.4
Yes, a lot		20.7 (7.3)	2072	7.1
**Disease among close relatives**				
No		11.4 (7.0)	17,853	60.4
Yes, a little		12.3 (6.9)	8127	25.7
Yes, partly		14.4 (7.5)	2842	9.1
Yes, a lot		17.5 (7.9)	1435	4.7
**Deaths among close relatives**				
No		11.8 (7.1)	24,581	81.2
Yes, a little		12.8 (7.0)	3235	10.6
Yes, partly		14.0 (7.2)	1293	4.3
Yes, a lot		16.2 (8.0)	1152	3.9
**Other types of distress**				
No		11.5 (7.0)	26,531	87.0
Yes, a little		14.2 (6.9)	1913	6.4
Yes, partly		17.2 (7.0)	1004	3.6
Yes, a lot		20.6 (7.5)	812	3.0
**Perceived social support**				
Yes, always		10.2 (6.6)	18,374	59.2
Yes, mostly		13.7 (6.8)	7707	25.8
Yes, sometimes		17.4 (6.9)	2825	10.1
No, never or almost never		16.8 (7.9)	1334	4.9

^a^Weighted percentage

Stata/SE v16.1 (StataCorp, College Station, TX) was used to prepare the data and perform the descriptive and inferential analyses including dominance analysis using the community-contributed extension DOMIN [66].

## Results

### Descriptive analyses

The mean perceived stress level in the population was 12.2 with a range from 0 to 40 (Table 1). The highest stress levels were found among women, 16–24-year-olds, respondents with an ethnic background other than Danish, respondents with a low educational attainment and respondents who were students or outside the labour marked (unemployed, receiving cash or sickness benefits, or receiving a disability pension). The three groups outside the labour market had the highest perceived stress levels of all subgroups.

The prevalence of stressors varied from 42.2% having been burdened by disease during the past 12 months to 13.0% having experienced other types of burdens (Table 2). Absence of perceived social support was found in 4.9%. For all stressors, the mean PSS increased with increasing intensity of the stressor. However, for perceived social support those who never or almost never had social support did not report a higher intensity of the stressor than those who sometimes had social support.

### Main findings

**Fig. 1:**
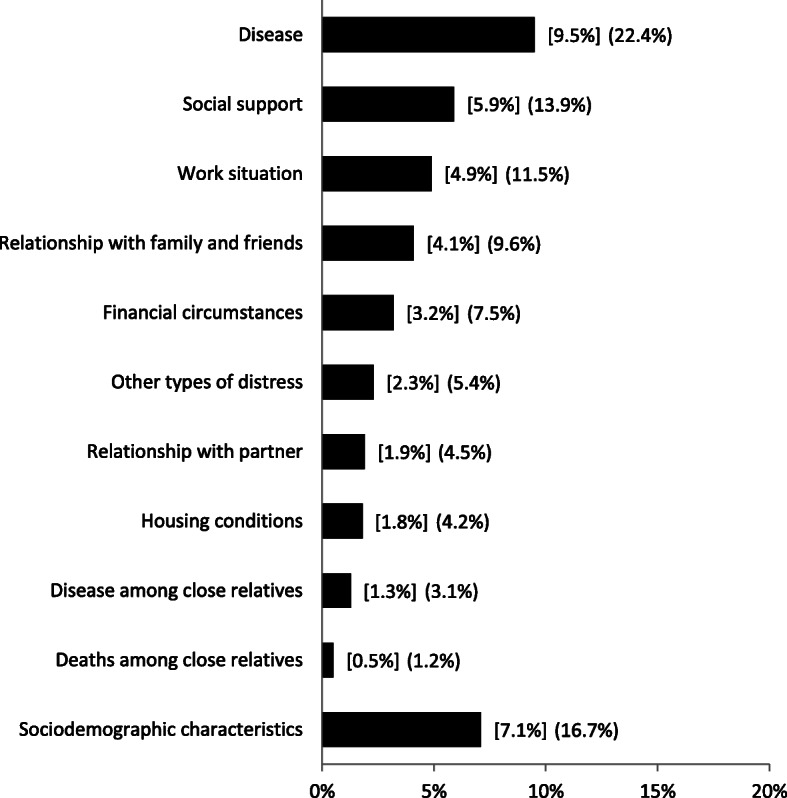
Adjusted dominance analyses (“dominance profiles”) for all respondents (*N* = 29,541). Rank of stressors and social support based on average increase in total variance explained by the model (R^2^) when adding a variable to all possible sub-models. Contribution to total variance is indicated next to the bar in absolute and relative (in parentheses) terms. Sociodemographic characteristics were included in all models

Work- and non-work-related stressors along with perceived social support explained 42.5% of the variance in overall perceived stress level in the adjusted analysis (Table 3 and Fig. 1). The three explanatory variables explaining most of the variance in PSS were disease (9.5%), perceived social support (5.9%) and work situation (4.9%) (Table 3 and Fig. 1). Adding the non-work-related stressors and perceived social support to the work-related stressor increased the explained variance from 12.1% (work situation 4.9% and sociodemographic characteristics 7.2%) to 42.5%.

**Table 3:** Unadjusted and adjusted dominance analyses (“dominance profiles”) for all respondents and subgroups stratified by gender, age, educational attainment and employment status (*N* = 29,860). Explanatory variables contributing with more than 10% of the adjusted *R*^2^ are stated in bold

	Unadjusted ***R***^**2**^	Adjusted ***R***^**2**^	Adjusted rank and ***R***^**2**^ of stressors and perceived social support	
**All**^**a**^	38.7% (*N* = 29,860)	42.5% (*N* = 29,541)	**1. Disease (9.5%)**	6. Other types of distress (2.3%)
			**2. Perceived social support (5.9%)**	7. Relationship with partner (1.9%)
			**3. Work situation (4.9%)**	8. Housing conditions (1.8%)
			4. Relationship with family and friends (4.1%)	9. Disease among close relatives (1.3%)
			5. Financial circumstances (3.2%)	10. Deaths among close relatives (0.5%)
**Gender**^**b**^				
Men	39.1% (*N* = 13,903)	41.9% (*N* = 13,770)	**1. Disease (10.0%)**	6. Other types of distress (2.3%)
			**2. Perceived social support (5.9%)**	7. Housing conditions (2.2%)
			**3. Work situation (5.4%)**	8. Relationship with partner (1.9%)
			4. Relationship with family and friends (4.0%)	9. Disease among close relatives (1.3%)
			5. Financial circumstances (3.2%)	10. Deaths among close relatives (0.5%)
Women	38.2% (*N* = 15,957)	42.1% (*N* = 15,771)	**1. Disease (9.2%)**	6. Other types of distress (2.5%)
			**2. Perceived social support (6.0%)**	7. Relationship with partner (1.9%)
			**3. Work situation (4.6%)**	8. Housing conditions (1.6%)
			**4. Relationship with family and friends (4.2%)**	9. Disease among close relatives (1.4%)
			5. Financial circumstances (3.2%)	10. Deaths among close relatives (0.5%)
**Age**^**c**^				
16–24	38.4% (*N* = 3302)	42.8% (*N* = 3272)	**1. Disease (6.7%)**	6. Other types of distress (3.0%)
			**2. Relationship with family and friends (6.3%)**	7. Housing conditions (1.9%)
			**3. Perceived social support (5.3%)**	8. Relationship with partner (1.5%)
			4. Work situation (3.6%)	9. Disease among close relatives (1.2%)
			5. Financial circumstances (3.6%)	10. Deaths among close relatives (0.1%)
25–64	43.1% (*N* = 18,447)	44.8% (*N* = 18,271)	**1. Disease (10.0%)**	6. Other types of distress (2.6%)
			**2. Work situation (6.9%)**	7. Housing conditions (2.1%)
			**3. Perceived social support (6.4%)**	8. Relationship with partner (2.1%)
			4. Relationship with family and friends (4.0%)	9. Disease among close relatives (1.3%)
			5. Financial circumstances (3.8%)	10. Deaths among close relatives (0.5%)
≥ 65	29.3% (*N* = 8111)	34.0% (*N* = 7998)	**1. Disease (10.9%)**	6. Other types of distress (1.3%)
			**2. Perceived social support (5.4%)**	7. Financial circumstances (1.2%)
			3. Relationship with family and friends (3.0%)	8. Housing conditions (1.1%)
			4. Relationship with partner (2.1%)	9. Deaths among close relatives (0.8%)
			5. Disease among close relatives (1.6%)	10. Work situation (0.3%)
**Educational attainment**^**d**^				
Low	36.6% (*N* = 4337)	38.7% (*N* = 4337)	**1. Disease (10.2%)**	6. Work situation (2.0%)
			**2. Perceived social support (5.6%)**	7. Relationship with partner (1.8%)
			3. Relationship with family and friends (3.7%)	8. Disease among close relatives (1.6%)
			4. Financial circumstances (2.6%)	9. Housing conditions (1.6%)
			5. Other types of distress (2.4%)	10. Deaths among close relatives (0.7%)
Medium	40.3% (*N* = 16,766)	42.6% (*N* = 16,766)	**1. Disease (10.3%)**	6. Other types of distress (2.1%)
			**2. Perceived social support (6.5%)**	7. Relationship with partner (1.9%)
			**3. Work situation (5.0%)**	8. Housing conditions (1.9%)
			4. Relationship with family and friends (4.2%)	9. Disease among close relatives (1.3%)
			5. Financial circumstances (3.6%)	10. Deaths among close relatives (0.5%)
High	39.8% (*N* = 8438)	41.9% (*N* = 8438)	**1. Disease (8.2%)**	6. Other types of distress (3.0%)
			**2. Work situation (7.4%)**	7. Relationship with partner (2.0%)
			**3. Perceived social support (5.1%)**	8. Housing conditions (2.0%)
			4. Relationship with family and friends (4.5%)	9. Disease among close relatives (1.4%)
			5. Financial circumstances (3.2%)	10. Deaths among close relatives (0.3%)
**Employment status**^**a**^				
Students	37.0% (*N* = 2535)	41.6% (*N* = 2526)	**1. Disease (6.8%)**	6. Other types of distress (3.3%)
			**2. Perceived social support (6.0%)**	7. Housing conditions (1.7%)
			**3. Relationship with family and friends (6.0%)**	8. Relationship with partner (1.7%)
			**4. Financial circumstances (4.3%)**	9. Disease among close relatives (0.8%)
			5. Work situation (3.4%)	10. Deaths among close relatives (0.4%)
Employed	36.1% (*N* = 16,167)	38.4% (*N* = 16,068)	**1. Work situation (9.0%)**	6. Financial circumstances (2.3%)
			**2. Perceived social support (6.1%)**	7. Other types of distress (1.9%)
			**3. Disease (6.0%)**	8. Housing conditions (1.5%)
			4. Relationship with family and friends (3.2%)	9. Disease among close relatives (1.4%)
			5. Relationship with partner (2.5%)	10. Deaths among close relatives (0.2%)
Unemployed	43.6% (*N* = 539)	46.1% (*N* = 536)	**1. Disease (10.2%)**	6. Relationship with partner (2.7%)
			**2. Work situation (7.1%)**	7. Housing conditions (2.5%)
			**3. Financial circumstances (5.4%)**	8. Other types of distress (1.4%)
			**4. Relationship with family and friends (4.7%)**	9. Disease among close relatives (0.7%)
			5. Perceived social support (4.2%)	10. Deaths among close relatives (0.4%)
Cash or sickness benefits	47.2% (*N* = 1401)	51.8% (*N* = 1362)	**1. Disease (13.8%)**	6. Work situation (3.3%)
			**2. Relationship with family and friends (6.2%)**	7. Housing conditions (2.4%)
			**3. Financial circumstances (5.3%)**	8. Disease among close relatives (2.1%)
			4. Perceived social support (4.4%)	9. Relationship with partner (1.4%)
			5. Other types of distress (3.8%)	10. Deaths among close relatives (0.4%)
Disability pension	38.7% (*N* = 1029)	42.6% (*N* = 986)	**1. Disease (8.4%)**	6. Housing conditions (2.6%)
			**2. Perceived social support (6.8%)**	7. Deaths among close relatives (2.1%)
			**3. Relationship with family and friends (5.8%)**	8. Disease among close relatives (1.9%)
			4. Other types of distress (3.2%)	9. Relationship with partner (1.5%)
			5. Financial circumstances (3.0%)	10. Work situation (1.4%)
Early retirement pension	41.4% (*N* = 714)	43.9% (*N* = 712)	**1. Disease (11.7%)**	6. Other types of distress (2.2%)
			**2. Perceived social support (9.6%)**	7. Work situation (2.0%)
			**3. Relationship with family and friends (4.9%)**	8. Relationship with partner (1.4%)
			4. Housing conditions (2.9%)	9. Disease among close relatives (1.3%)
			5. Financial circumstances (2.9%)	10. Deaths among close relatives (1.2%)
Retirement pension	29.6% (*N* = 7299)	33.3% (*N* = 7187)	**1. Disease (11.0%)**	6. Other types of distress (1.4%)
			**2. Perceived social support (5.6%)**	7. Financial circumstances (1.2%)
			3. Relationship with family and friends (3.2%)	8. Housing conditions (1.2%)
			4. Relationship with partner (2.4%)	9. Deaths among close relatives (0.8%)
			5. Disease among close relatives (1.6%)	10. Work situation (0.2%)

^a^Adjusted for gender, age, ethnic background and educational attainment

^b^Adjusted for age, ethnic background and educational attainment

^c^Adjusted for gender, ethnic background and educational attainment

^d^Adjusted for gender, age and ethnic background

### Stratified analyses

The explanatory variables explained 41.9 and 42.1% of the variance in PSS in men and women, respectively (Table 3). In both men and women, the most important explanatory variables were disease, perceived social support and work situation. In the analyses stratified by age group, the explanatory variables explained 42.8, 44.8 and 34.0% of the variance in PSS, respectively (Table 3). In the young age group (16–24 years), the most important explanatory variables were disease, relationship with family and friends and perceived social support. In the middle-aged group (25–64 years), the most important explanatory variables were disease, work situation and perceived social support. In the elderly group (65 years and above), the most important explanatory variables were disease and perceived social support.

In the analyses stratified by educational attainment, the explanatory variables explained 38.7% of the variance in PSS in the low, 42.6% in the medium and 41.9% in the high educational groups (Table 3). In the low educational attainment group, the most important explanatory variables were disease and perceived social support. In the medium educational attainment group, the most important explanatory variables were disease, perceived social support and work situation. Finally, in the high educational attainment group, the most important explanatory variables were disease, work situation and perceived social support.

In the analyses stratified by employment status, the explanatory variables explained 41.6% for students, 38.4% for employed, 46.1% for unemployed, 51.8% for respondents receiving cash or sickness benefits, 42.6% for respondents receiving disability pension, 43.9% for respondents receiving early retirement pension and 33.3% for respondents receiving retirement pension (Table 3). The top three explanatory variables included disease in all seven subgroups and perceived social support in five subgroups. Among students, the most important explanatory variables were disease, perceived social support, relationship with family and friends and financial circumstances. Among employed respondents, the most important explanatory variables were work situation, perceived social support and disease. Among unemployed respondents, it was disease, work situation, financial circumstances and relationship with family and friends. Among those receiving cash or sickness benefits, it was disease, relationship with family and friends and financial circumstances. Among those receiving disability pensions, it was disease, perceived social support and relationship with family and friends. Furthermore, among those receiving early retirement pensions it was disease, perceived social support and relationship with family and friends. Finally, among those receiving retirement pensions, it was disease and perceived social support.

## Discussion

### Main findings and stratified analyses

We applied a comprehensive approach to stress, investigating the relative importance of work- and non-work-related stressors and perceived social support on overall perceived stress in a representative adult population and in sociodemographic subgroups. As recommended in a number of previous studies [3, 8, 11, 31, 32], we investigated both work- and non-work-related explanatory variables of stress. Most noteworthy, the overall perceived stress level was statistically explained by a combination of work- and non-work-related perceived stressors and perceived social support. The most important explanatory variables of perceived stress were disease, perceived social support and work situation. In fact, including non-work-related stressors and perceived social support along with work-related stressors more than tripled the explained variance in perceived stress compared with work-related stressors alone.

The stratified analyses (including different subgroups) resulted in slightly varying “dominance profiles” of perceived stress. However, disease was the most important explanatory variable of perceived stress in 14 of the 15 subgroups. Perceived social support was among the top three explanatory variables in 13 subgroups, and work situation was among the top three explanatory variables in seven subgroups (only relevant in eight subgroups). Other important explanatory variables in the stratified analyses were relationship with family and friends, and financial circumstances. Work situation was the single most important explanatory variable in the employed group, and the second most important explanatory variable in the unemployed group. However, even in the employed group adding non-work-related stressors and perceived social support tripled the explained variance.

Importantly, although a specific stressor explains only a small part of the overall variation in stress level, it may well play an important role in combination with other stressors [32]. This may also hold true for stressors with low contribution to the explained variance in the dominance analyses since stressors rarely occur in isolation. The combined effect of several stressors can therefore have a negative effect on the stress level. In addition, a stressor can be part of a chain of stressors triggered by a primary stressor (*stress proliferation*), or there may be a spill over of stressors across roles or life domains (conflicts between work and family, etc.) [10], which also demonstrates the importance of examining multiple stressors simultaneously.

Our results are in accordance with the Stress Process Model, which finds that stressors combine to produce strain [10], and with previous studies reporting that combinations of stressors from both work and non-work domains contribute to mental health [3, 8] and perceived stress in particular [11, 31, 32, 67, 68]. Our results add important knowledge to the vast majority of studies on stress that have focused on different aspects of the work situation [2, 5, 13, 20, 62, 69, 70]. A review concluded that since the majority of studies do not include non-work domains, they may overlook the contribution from non-work predictors [3]. Furthermore, Marchand and colleagues concluded that including both work and non-work domains is necessary to avoid erroneous conclusions about the relationship between work and mental health [8]. For example, recent years have seen an increased focus on the implications of managing work and family caregiving roles (child caregiving, elder caregiving or “sandwiched” caregiving) with a special focus on gender differences [71]. In line herewith, our results – both at population level and in subgroups – indicate that in order to understand the complex interaction of stressors, both non-work-related stressors and perceived social support should be addressed along with the work situation. Thus, our results are in favour of a comprehensive approach to understanding the relationship between perceived stress, work- and non-work-related stressors and perceived social support, and therefore also of developing public health strategies and interventions aiming at reducing perceived stress at the population level and in high-risk groups.

### Explanatory variables

 The three most important explanatory variables of perceived stress in this study were disease, perceived social support and work situation. An American study of older adults identified loneliness, neighbourhood and financial strain as particularly important predictors of perceived stress [31]. Different groups of respondents may have different “dominance profiles”of perceived stress, which is indicated in our study.

Disease as a stressor has received far less attention than stress as a risk factor for developing diseases. Even so, an understanding of the pathway from disease to stress is beginning to emerge [72]. This pathway includes loss of control and uncertainty leading to a feeling of threat, which may lead to hopelessness and stress if coping mechanisms are inadequate. This may be the reason why disease is the most important explanatory variable of overall perceived stress in our study. Four pathways from chronic disease to stress have been suggested [72]: 1) Disease often causes pain, reduces physical and mental functioning and can be life threatening, making it difficult to meet the demands of everyday life. 2) The process of diagnosis and treatment in itself gives rise to concern and uncertainty and can be both financially burdensome and time consuming. 3) Patients may have concerns whether possible harms outweigh potential benefits of treatment and may experience adverse drug interactions if treated for multiple conditions. 4) Treatment can disrupt social roles and relationships and cause lower self-efficacy and self-worth, thereby affecting mental well-being negatively.

Lack of social support is a well-known predictor of poor mental health [28, 33, 34, 73]. Non-work social support can come from a number of sources including a partner, close relatives, friends, neighbours, etc. A study of workplace-perceived social support finds that job performance was strengthened by perceived supervisor and co-worker support [74]. Our results are in line with a Danish study reporting that a low level of private-life social support increases the risk of symptoms of depression [73] and a review concluding that social support is associated with mental health [34]. Even though we do not distinguish between work- and non-work-related social support, our results are in accordance with a recent meta-analysis showing that a low level of support at work increases the risk of stress-related mental disorders [2]. However, it remains to be elucidated how social support produces positive mental health outcomes [36].

The work situation is a well-known stressor [2, 4, 5], which is also confirmed in our study. Different aspects of the work situation have been identified as predictors of stress, e.g. effort-reward imbalance, high job demands, organisational justice, social support, emotional demands and decision authority [2]. A study reports that stress from work-related strain may be transferred from parents to children [75]. This may also hold true for transfer to one’s partner.

Although The Stress Process Model has served as a paradigm for stressor research since the 1980s, a lack of understanding remains of how work- and non-work-related stressors, coping, social support and personal resources interact with each other [25]. Gaining a deeper understanding of this interaction is mandatory to develop effective strategies against stress encompassing people’s entire life situation [31, 32]. An American study found that similar combinations of chronic stressors and life events may result in different levels of perceived stress (*multifinality*) and that different combinations of stressors may produce similar levels of perceived stress (*equifinality*) [32].

### Strengths and limitations

The present study has several strengths. We use a combination of self-reported and register data from a representative population sample, the sample size is large and the response rate was reasonably high. Register data are of high validity and accuracy. Overall perceived stress was assessed using the PSS, which has satisfying psychometric properties. Explanatory variables of perceived stress covered a wide range of sources. Furthermore, the dominance analysis allows us to rank the importance and contribution of explanatory variables and eases the interpretation of several regression coefficients simultaneously [61, 76]. Finally, data are weighted, and analyses are adjusted for a number of factors, which is expected to reduce residual confounding.

The study also has limitations. Although the study has a theoretical framing, it is not possible to draw causal conclusions from its results due to the cross-sectional nature of the data. Instead, we investigate the ability to statistically explain the variation in perceived stress levels using perceived stressors and perceived social support. As such, the study can be seen as part of a more comprehensive research effort to develop and refine explanatory models that can ultimately be tested in a causal setup (cf. the distinction between explanatory and predictive modelling presented by Shmueli [77]). In this study we focused on perceived stressors within nine life domains. Although comprehensive, our model does not include several areas of central importance to human existence and relevant to our understanding of stress and health. Examples of such areas are rest and restitution, loneliness, sleep, nutrition, physical activity and sexual relationships. We therefore need to develop richer, more complex models to capture even more aspects of the stress process.

The major risk with conducting a dominance analysis is not identifying and using the correct model [60]. Furthermore, omission of variables that belong in the model can bias comparisons. In this study both the model and the variables are carefully chosen. The dominance analysis is based on a multiple linear regression model, and the explanatory variables are chosen according to our theoretical framework.

Non-respondents are more likely to be 16–24-years and of an ethnic background other that Danish. Therefore, non-response bias cannot be excluded. Perceived stress, stressors and social support are assessed within different time frames, which may weaken the variance explained by the explanatory variables. This may partly explain why the dominance analyses explain only around 40% of the total variance. In the 16–24-year-old group, educational level is based on assumptions about future educational level resulting in a larger proportion having a low and medium-high educational level compared with the 25–64-year-old group.

## Conclusions

The results of this population-based study demonstrate that the overall perceived stress level can be statistically explained by a combination of work- and non-work-related stressors and perceived social support. This is the case both at population level and in stratified subgroups. The three most important explanatory variables of perceived stress in the dominance analysis are disease, perceived social support and work situation. Hence, the study indicates that we should address a variety of stressor domains rather than focus on a single domain like work-related stressors. Thus, our results point towards a return to a comprehensive approach to stress as suggested by Pearlin and colleagues [10].

Work situation is the most important explanatory variable of perceived stress in the employed group. However, even in this group, adding the non-work-related explanatory variables increased the explained variance noticeably, suggesting that work-related stressors only capture part of the total amount of stressors affecting perceived stress. Thus, even among those employed, stress preventions should not focus solely on work-related stressors.

This study provides novel insight into the determinants of overall perceived stress level applicable to public health strategies aiming to reduce stress both at population level and in high-risk subgroups. Stratified subgroup analyses revealed varying “dominance profiles” profiles suggesting a need for target group-specific stress reduction strategies.

## Supplementary Information

**Additional file 1: Correlation table.** Pairwise correlation between perceived stressors, perceived social support and perceived stress (PSS) (*significance level *p* = 0.01, Bonferroni adjusted).

## Data Availability

The datasets used and analysed during the present study are available from the corresponding author on reasonable request.
