# Supplementary material for: The relative importance of work-related and non-work-related stressors and perceived social support on global perceived stress in a cross-sectional population-based sample
Source: BMC Public Health. 2021 Mar 19;21:543. doi: 10.1186/s12889-021-10594-2 (PMC7980655; doi:10.1186/s12889-021-10594-2)
Supplement: Supplementary file 1 — Additional file 1: Correlation table. Pairwise correlation between perceived stressors, perceived social support and perceived stress (PSS) (*significance level p = 0.01, Bonferroni adjusted). [file 12889_2021_10594_MOESM1_ESM.docx]

# Supplementary material

## Title

The relative importance of work-related and non-work-related stressors and perceived social support on global perceived stress in a cross-sectional population-based sample

## Authors

Jes Bak Sørensen^1^, Mathias Lasgaard^1^, Morten Vejs Willert^2^, Finn Breinholt Larsen^1^

^1^ DEFACTUM, Central Denmark Region, Denmark.

^2^ Department of Occupational Medicine, Danish Ramazzini Centre, Aarhus University Hospital, Denmark.

Corresponding author: Jes Bak Sørensen, DEFACTUM, Central Denmark Region, Olof Palmes Alle 15, DK-8200 Aarhus N, Denmark. Email [jesbso@rm.dk](mailto:jesbso@rm.dk).

Correlation table. Pairwise correlation between perceived stressors, perceived social support and perceived stress (PSS) (*significance level p=0.01, Bonferroni adjusted).

|  | Financial circumstances | Housing conditions | Work situation | Relationship with partner | Relationship with family and friends | Disease | Disease among close relatives | Deaths among close relatives | Other types of distress | Perceived social support | PSS |
| --- | --- | --- | --- | --- | --- | --- | --- | --- | --- | --- | --- |
| Financial circumstances | 1.0000_ |  |  |  |  |  |  |  |  |  |  |
| Housing conditions | 0.4791* | 1.0000_ |  |  |  |  |  |  |  |  |  |
| Work situation | 0.4062* | 0.3196* | 1.0000_ |  |  |  |  |  |  |  |  |
| Relationship with partner | 0.2416* | 0.2343* | 0.2202* | 1.0000_ |  |  |  |  |  |  |  |
| Relationship with family and friends | 0.2765* | 0.2919* | 0.2588* | 0.3439* | 1.0000_ |  |  |  |  |  |  |
| Disease | 0.2078* | 0.1662* | 0.1994* | 0.1289* | 0.2105* | 1.0000_ |  |  |  |  |  |
| Disease among close relatives | 0.1101* | 0.1165* | 0.1000* | 0.1621* | 0.1914* | 0.1959* | 1.0000_ |  |  |  |  |
| Deaths among close relatives | 0.0538* | 0.0734* | 0.0343* | 0.0630* | 0.0940* | 0.1158* | 0.2970* | 1.0000_ |  |  |  |
| Other types of distress | 0.1816* | 0.1765* | 0.1618* | 0.1460* | 0.2081* | 0.2053* | 0.1604* | 0.1079* | 1.0000_ |  |  |
| Perceived social support | 0.1582* | 0.1595* | 0.1270* | 0.2088* | 0.2672* | 0.1732* | 0.0770* | 0.0467* | 0.1340* | 1.0000_ |  |
| PSS | 0.3561* | 0.2980* | 0.3453* | 0.2569* | 0.3653* | 0.4301* | 0.1991* | 0.1293* | 0.2663* | 0.3460* | 1.0000 |
